# High incidence of asymptomatic leptospirosis among urban sanitation workers from Kota Kinabalu, Sabah, Malaysian Borneo

**DOI:** 10.1038/s41598-020-76595-0

**Published:** 2020-11-10

**Authors:** Mohammad Saffree Jeffree, Daisuke Mori, Nur Athirah Yusof, Azman Bin Atil, Khamisah Awang Lukman, Rafidah Othman, Mohd Rohaizat Hassan, Lela Suut, Kamruddin Ahmed

**Affiliations:** 1 Department of Community and Family Medicine, Faculty of Medicine and Health Sciences, Universiti Malaysia Sabah, 88400 Kota Kinabalu, Sabah Malaysia; 2 Borneo Medical and Health Research Centre, Faculty of Medicine and Health Sciences, Universiti Malaysia Sabah, 88400 Kota Kinabalu, Sabah Malaysia; 3 Department of Pathobiology and Medical Diagnostics, Faculty of Medicine and Health Sciences, Universiti Malaysia Sabah, 88400 Kota Kinabalu, Sabah Malaysia; 4 Biotechnology Research Institute, Universiti Malaysia Sabah, 88400 Kota Kinabalu, Sabah Malaysia; 5 Borneo Marine Research Institute, Universiti Malaysia Sabah, 88400 Kota Kinabalu, Sabah Malaysia; 6 Department of Community Health, Faculty of Medicine, Universiti Kebangsaan Malaysia, 56000 Cheras, Kuala Lumpur, Malaysia; 7 Faculty of Medicine and Health Sciences, Universiti Malaysia Sarawak, 94300 Kota Samarahan, Sarawak Malaysia

**Keywords:** Microbiology, Bacteria, Clinical microbiology, Pathogens, Epidemiology

## Abstract

Leptospirosis is a public health challenge in Sabah State of Malaysian Borneo. Rapid urbanization, rural-to-urban migration, and undocumented immigration in Sabah have increased the pressure on the urban garbage disposal system. Rodents and other small animals thrive under these conditions. We hypothesized that urban sanitation workers would be at risk of developing leptospirosis. In total, 303 urban sanitation workers with a mean age of 42.6 years were enrolled in this study. The serum samples collected from these workers were subjected to the microscopic agglutination test (MAT), PCR and nucleotide sequencing of the amplicons to confirm the presence of *Leptospira.* The phylogenetic analysis using the neighbor joining method was performed to assess whether they were pathogenic. In this study 43.8% (133/303) of the samples were MAT-seropositive and among them, 29 (21.8%) were positive by PCR. Nucleotide sequencing of the amplicons confirmed the presence of *Leptospira.* Phylogenetic analysis showed that our strains belonged to the pathogenic group of *Leptospira.* A high proportion of urban sanitation workers were seropositive for leptospirosis, and a considerable number were PCR positive for *Leptospira,* thereby indicating asymptomatic infections. Further research is needed to confirm whether this is a transient phenomenon or antibiotic therapy is required.

## Introduction

Leptospirosis is the most common emerging zoonotic disease globally and it is expected to become more prominent worldwide because of climate change and the growing urban population^1^. Although leptospirosis is an underreported disease, 1.03 million people are infected worldwide and it causes 58,900 deaths every year^2^. Most of these deaths occur in tropical countries because the incidence of leptospirosis in the tropics is approximately 10 times higher than that in temperate regions^3^. Malaysia is a tropical country that has undergone rapid economic growth during recent decades and large numbers of people have moved into urban areas. At present, 76% of the total population lives in urban areas. Leptospirosis is an endemic notifiable disease in Malaysia, and it has been detected at alarming levels in different states throughout the country^4^.

Sabah is one of the 13 states of Malaysia and one of the two states located on the island of Borneo. According to data provided by the Sabah State Health Department, the incidence of leptospirosis is 4.07–26.68 per 100,000 people in the state. In the year 2000, a widely reported outbreak of leptospirosis occurred in Sabah that affected 304 athletes from 26 countries after they participated in the Eco-challenge race^5^. In 1999, another outbreak affected 46 patients after swimming in a creek in Beaufort district, Sabah^6^. Analyses have shown that the numbers of leptospirosis cases that occurred during outbreaks in Sabah only comprised a modest proportion of the total disease incidence, and most of these were related to multiple isolated cases reported from every district in Sabah. The high prevalence of leptospirosis is related to occupational activities, such as close contact with animals or exposure to water, mud, soil, vegetation, and waste contaminated with urine from animals with a high risk of infection^7,8^.

Among the infected animals, *Leptospira* spp. are most often associated with rodents, including the species that are frequently found in urban areas^9^. The prevalence of leptospirosis in urban habitats occupied by wildlife is higher than that found in natural environments, and this trend appears to be particularly significant for rodents^10^. In cities, the rodent populations are often larger and denser than those found in natural environments, which can lead to higher rates of contact with people and affect the risk of human diseases^9^. Leptospirosis is recognized as a hazard for certain occupations with an increased risk of exposure to infected animals, such as agricultural workers, sewage workers, military personnel, veterinarians and animal handlers^5,11^. However, the occupational risk of leptospirosis in Sabah remains unknown.

Urban sanitation workers are among the occupations that may have high risk of leptospirosis in Malaysia. Kota Kinabalu is the capital of Sabah and it has a population of 553,900. Rapid urbanization, migration of the rural population, an influx of undocumented immigrants, increased garbage disposal, and tropical weather have facilitated exponential increases in the rodent population in Kota Kinabalu, thereby exposing urban sanitation workers to *Leptospira*^12^*.* Pathogenic *Leptospira* species can cause a wide range of diseases in humans, where the complications can range from severe, such as Weil’s disease and pulmonary hemorrhagic syndrome, to mild flu-like symptoms^13^. Thus, we hypothesized that urban sanitation workers in Kota Kinabalu might be repeatedly exposed to *Leptospira* and have a high rate of seropositivity for *Leptospira,* and that some might be experiencing asymptomatic leptospirosis. However, no previous studies have investigated this important public health issue in Sabah. Therefore, the present study aimed to determine the consequences of repeated exposure to *Leptospira* among sanitation workers in Kota Kinabalu.

## Methods

### Study area, population, and ethics statement

A cross-sectional analysis was conducted in March 2017 among urban sanitary workers from Kota Kinabalu, Sabah, Malaysia. Only people who had worked outdoor environments for more than 6 months were enrolled in this study. Ethical clearance (JKEtika: 4/16[1]) was obtained from the Ethics Committee of the Faculty of Medicine and Health Sciences, Universiti Malaysia Sabah, Kota Kinabalu, Sabah, Malaysia (UMS). All methods were performed in accordance with the relevant guidelines and regulations. Written informed consent was obtained from all participants. We collected 10 mL of venous blood from each participant and separated the serum. The serum samples were transported by cold chain to the laboratory at UMS and Kota Kinabalu Public Health Laboratory. All of the serum samples were stored at – 80 °C until they were analyzed.

### Determination of seroprevalence using microscopic agglutination test (MAT)

The MAT was conducted according to standard methods at Kota Kinabalu Public Health Laboratory to determine the antibody titers against *Leptospira* serovars. It is the state reference laboratory for MAT to diagnose leptospirosis. Serum samples were serially diluted in microtiter plates, and to each well were added 20 live *Leptospira* serovars recommended by the Institute of Medical Research Malaysia (*L. meyeri* serovar Melaka, *L. kemamanensis* serovar Terengganu, *L. sarikeinsis* serovar Sarawak, *L. interrogans* serovar Copenhageni, *L. borgpetersenii* serovar Hardjobovis, *L. borgpetersenii* serovar Lai, *L. interrogans* serovar Australis, *L. interrogans* serovar Autumnalis, *L. interrogans* serovar Bataviae, *L. interrogans* serovar Canicola, *L. weilii* serovar Celledoni, *L. krischneri* serovar Grippotyphosa, *L. borgpetersenii* serovar Hardjoprajitno, *L. interrogans* serovar Icterohaemorrhagiae, *L. borgpetersenii* serovar Javanica, , *L. interrogans* serovar Pyrogenes, *L. borgpetersenii* serovar Terrasovi, *L. interrogans* serovar Djasiman, *L. biflexa* serovar Patoc and, *L. interrogans* serovar Pomona). The Institute of Medical Research is the national reference laboratory, the recommended serovars are based on the background exposure and seroprevalence of leptospirosis in the country. The plates were then incubated for 2 h at 30 °C and examined by dark-field microscopy. A mixture was considered positive when it exhibited 50% agglutination and 50% of the cells were free compared with the control culture. MAT titers were reported as the reciprocal of the number of dilutions that still agglutinated 50% of the live bacterial antigen. A titer of ≥ 1:50 was taken as reactive to that serovar.

### Determination of Leptospira by PCR

DNA was extracted from serum samples using a DNeasy Blood Tissue Kit (Qiagen, Hilden, Germany) according to the manufacturer’s instructions. To determine whether the serum samples from the urban sanitary workers contained *Leptospira,* nested PCR assays were performed using GoTaqGreen Master Mix (Promega Corporation, Madison, WI, USA) and primers generated from the flagellin b gene of *Leptospira*^14^. The first PCR primers were LflaB-F1 (5′-CTCACCGTTCTCTAAAGTTCAAC-3′) and L-flaB-R1 (5′- TGAATTCGGTTTCATATTTGCC-3′). The second PCR primers were L-flaB-F2 (5′-TGTGCACAAGACGATGAAAGC-3′) and L-flaB-R2 (5′-AACATTGCCGTACCACTCTG-3′).

Nucleotide sequencing was performed for the amplicons using a BigDye Terminator v3.1 Cycle Sequencing kit (Applied Biosystems, Gaithersburg, MD, USA). The purified amplicons were sequenced using an ABI3130 Genetic Analyzer (Applied Biosystems). The second primers were used for sequencing. All of the procedures were conducted according to the manufacturer’s instructions. PCR positive samples were taken as asymptomatic leptospirosis positive samples.

### Sensitivity, specificity, positive and negative predictive values of MAT for asymptomatic leptospirosis

The sensitivity, specificity, positive predictive value (PPV) and negative predictive value (NPV) of MAT titer (1:50) to identify asymptomatic cases as determined by PCR were calculated by using MedCalc statistical software (https://www.medcalc.org/calc/diagnostic_test.php).

### Phylogenetic analysis

Phylogenetic analysis was performed to determine the relatedness among our strains and pathogenic and intermediate pathogenic *Leptospira* strains. We also selected 30 newly reported species of *Leptospira* for phylogenetic analysis^15^. The nucleotide sequences of the flagellin b genes from pathogenic and intermediate pathogenic and newly reported *Leptospira *were retrieved from GenBank. Multiple sequence alignment was performed using CLUSTALW ver. 2^16^. Phylogenetic analyses were conducted with the neighbor-joining method using the software MEGA ver. 7. The branching patterns were evaluated statistically by bootstrap analyses with 1,000 replicates.

## Results

**Figure 1:**
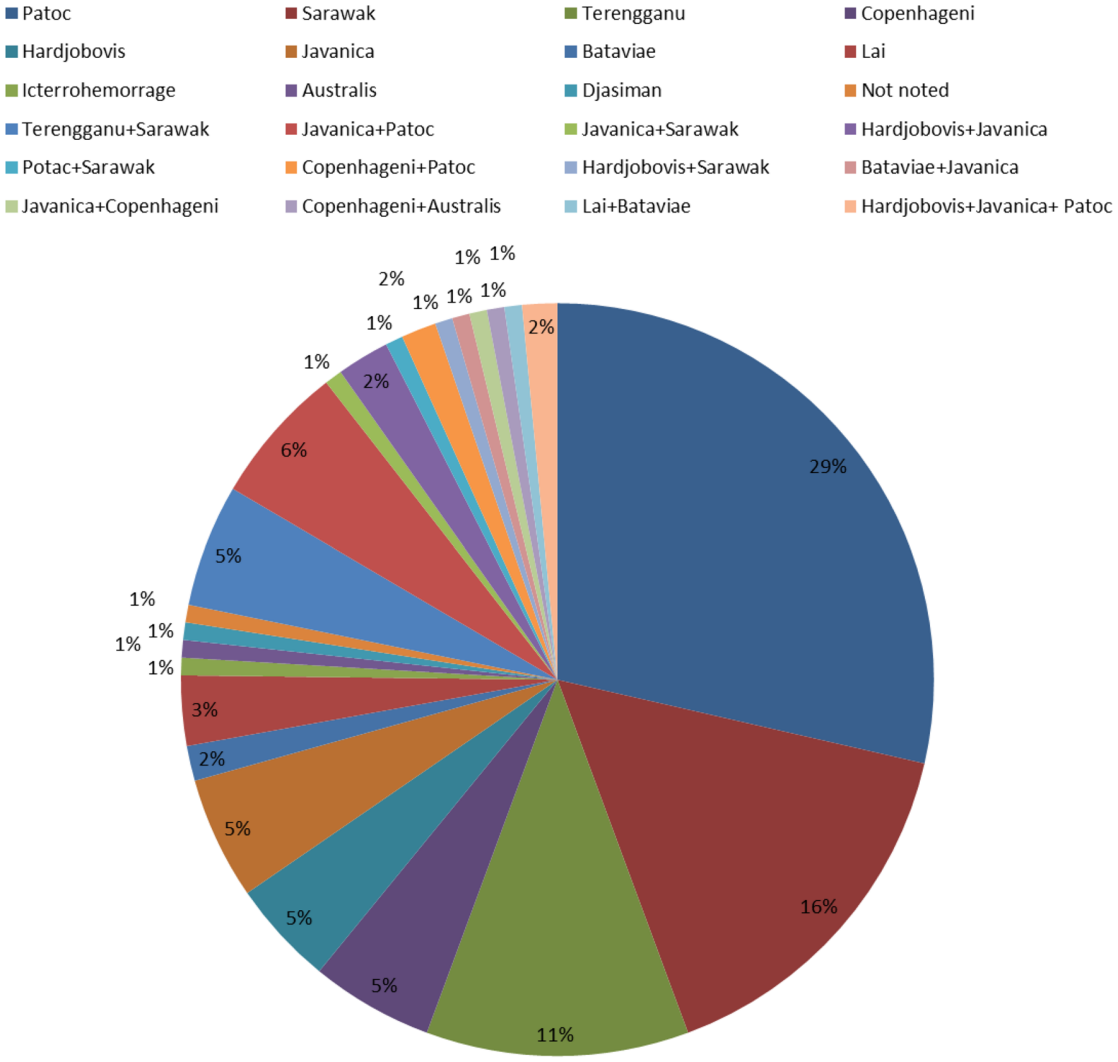
Distribution of reactive serovars in microscopic agglutination test (MAT) seropositive (> 1:50 as the cutoff value) samples represented as a pie chart. Colors representing different *Leptospira* serovars are indicated above the pie chart.

In total, 303 sanitation workers who fulfilled all of the criteria were enrolled in this study with 222 males (73.3%), 78 females (25.7%), and three participants with an unrecorded gender (1%). The male:female ratio was 2.8:1. The mean age was 42.6 years (range 23–60 years). With a cutoff value of ≥ 1:50, 43.8% (133/303) of the samples were seropositive for *Leptospira.* Among the 20 recommended *Leptospira* serovars used for MAT, only 11 were reactive with our serum samples. Among the 133 seropositive samples, 104 (78.2%) were reactive with one serovar, 27 (20.3%) were reactive with two serovars, and only two (1.5%) were reactive with three serovars. Antibodies to serovars Patoc, Sarawak, and Terengganu were detected in 56% of the serum samples that reacted to single serovars, and antibodies to serovars Terengganu + Sarawak and Javanica + Patoc were detected in 11% of the serum samples that reacted to two serovars (Fig. 1).

**Figure 2:**
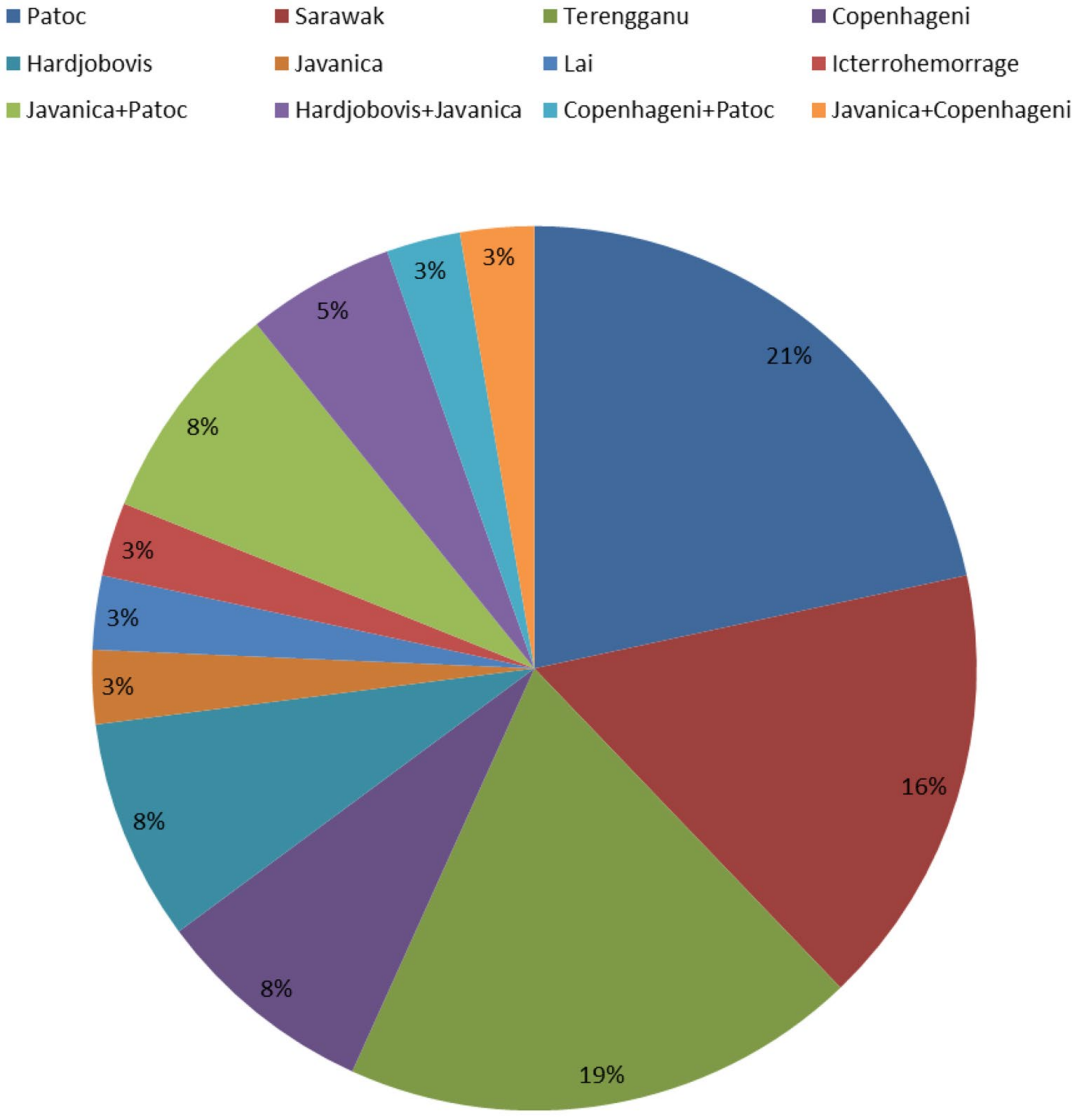
Distribution of reactive serovars detected by microscopic agglutination test (MAT) among samples that tested positive for *Leptospira* according to PCR represented as a pie chart. Colors representing different *Leptospira* serovars are indicated above the pie chart.

A 732-base pair amplicon generated by nested PCR indicated the presence of *Leptospira.* Among the 133 MAT-seropositive samples, 29 (21.8%) were positive by *Leptospira*-specific PCR. It may be mentioned here that PCR cannot distinguish between viable and nonviable organisms. Among the PCR-positive samples, Patoc, Sarawak, and Terengganu serovars accounted for 56% of the serum samples that reacted to single serovars in MAT, and Javanica + Patoc and Hardjobovis + Javanica accounted for 13% of the serum samples that reacted to two serovars in MAT (Fig. 2).

Compared to PCR the sensitivity, specificity, PPV, and NPV of MAT for the detection of asymptomatic leptospirosis were 100% [95% confidence intervals (CI) 88.1–100%], 62.0% (95% CI 56.0–67.8%), 21.8% (95% CI 19.3–24.5%), and 100%, respectively.

Nucleotide sequencing was successful for all of the PCR-positive samples and they were confirmed as *Leptospira* by aligning sequences using the Basic Local Alignment Search Tool (BLAST). All of the MAT-negative samples were also negative for *Leptospira* specific PCR.

**Figure 3:**
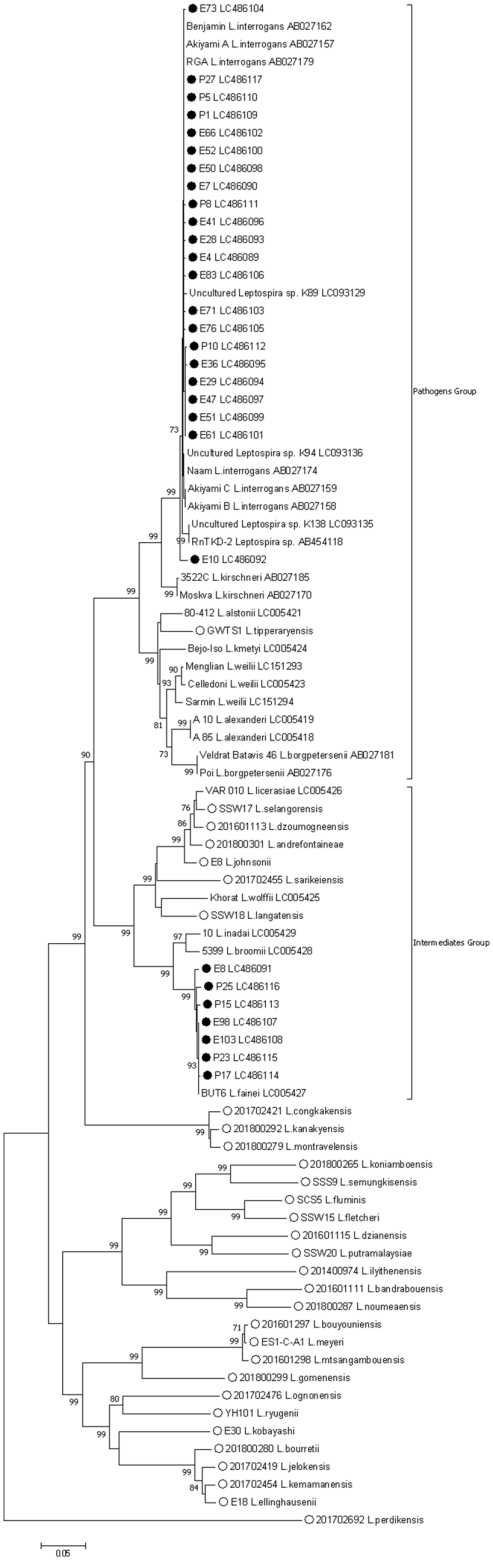
Phylogenetic tree constructed using the nucleotide sequences of the flagellin b gene in *Leptospira.* Nucleotide sequences of pathogenic, intermediate pathogenic, and recently reported new (open circle) strains were extracted from GenBank; our strains (closed circle) clustered with either pathogenic and intermediate pathogenic strains of *Leptospira.* Please note that generally the pathogenic potential of the newly reported *Leptospira* is unknown. Strains names are followed by GenBank accession numbers. The strains analyzed in this study are denoted by filled circles. The numbers adjacent to the nodes represent the bootstrap values, and values < 70% were omitted from this tree. The scale bar at the bottom indicates the genetic distance expressed as nucleotide substitutions per site.

Phylogenetic analysis showed that our strains belonged to the pathogenic group of *Leptospira* (Fig. 3). In particular, 21 belonged to a single cluster that mainly contained *Leptospira interrogans* strains. A single strain did not cluster with these strains. Seven of the strains belonged to the intermediate pathogenic group of *Leptospira* and they formed a single cluster with a *Leptospira fainei* strain detected in Japan.

## Discussion

In the present study, we determined that about 40% of the urban sanitation workers who were frequently exposed to the environment were seropositive for *Leptospira* and one-third of this population were PCR positive for *Leptospira,* but none complained of illness, thereby indicating that they had asymptomatic *Leptospira* infections. We did not assess fever or other symptoms of leptospirosis because the samples were collected during the workday, and thus we considered all participants to be healthy. Compared with urban sanitation workers in Kelantan (25.5%) and Selangor (34.8%) states, the seroprevalence of *Leptospira* was considerably higher in the present study^17,18^. The detection of DNA from pathogenic *Leptospira* species by PCR is one of the confirmatory tests employed for detecting leptospirosis^19^. In the present study, BLAST and phylogenetic analyses confirmed that pathogenic *Leptospira* isolates were detected by PCR. In addition to a positive PCR result, a fourfold increase in the antibody titer between acute and convalescent serum samples based on MAT or a high (≥ 1:400) MAT titer are confirmatory tests for leptospirosis^19^. Although these MAT criteria are recommended for the diagnosis of symptomatic leptospirosis, but there are no similar diagnostic criteria for asymptomatic infections. Clearly, PCR may be the most suitable test of choice for the diagnosis of asymptomatic cases. We found that the MAT titer was not related to the positivity by PCR in the diagnosis of asymptomatic leptospirosis. The MAT titer was > 1:50 for all of the PCR-positive samples, but not all samples with a MAT titer ≥ 1:50 were PCR-positive. However, all samples with MAT titer of < 1:50 were PCR negative for *Leptospira,* thereby indicating that MAT can be used to determine the absence of asymptomatic leptospirosis, but PCR is required to confirm the diagnosis of asymptomatic leptospirosis in cases where the MAT titer is > 1:50. Statistical analyses also support these findings. The MAT titer reflects the presence of both immunoglobulin G (IgG) and M (IgM) antibodies against *Leptospira*, and thus the MAT-positive cases may have exhibited high levels of IgG but not IgM, thereby indicating past exposure. These subjects may have been repeatedly exposed to *Leptospira,* so they might have exhibited higher levels of IgG in their serum.

The risk of infection after contact with a *Leptospira-*contaminated environment depends on the ability of bacteria to survive, persist and infect new hosts^20^. The factors that favor the environmental recycling and transmission of *Leptospira* species are not well understood^20–22^. However, the capacity of *Leptospira* for biofilm formation and cell aggregation might allow the maintenance of a sufficient concentration of bacteria to achieve an infection^23–26^.

Textbooks and other publications often mention the presence of asymptomatic leptospirosis, but its true frequency is unknown. Asymptomatic leptospirosis was reported based on the detection of IgM in subjects from an area with a previous *Leptospira* outbreak in Nicaragua^27^ but *Leptospira* was not detected by culture or molecular methods. In addition, human asymptomatic leptospirosis was reported from a rural area of Thailand^28^, where only one of 37 human samples was positive for *Leptospira* by PCR, and the study site was selected because human cases occurred 1–2 months previously. Indeed, *Leptospira* can be isolated from blood or cerebrospinal fluid several months after an acute episode^29^. Therefore, these studies might have detected the aftereffects of an acute episode of leptospirosis rather than asymptomatic infections. In a study conducted in the Seychelles, positive *Leptospira* PCR tests confirmed asymptomatic cases in 9% of the healthy adult males with manual occupations^29^. In general, it is accepted that humans can excrete *Leptospira* for weeks to months after an infection^30^; however, a recent study in the Peruvian Amazon found that asymptomatic individuals without serological evidence of recent exposure were shedding either pathogenic or intermediately pathogenic *Leptospira* in their urine^31^. Another study in India used PCR to find that *Leptospira* was excreted in the urine by 30.2% of the asymptomatic individuals investigated^32^. It is not known whether the excretion of *Leptospira* in urine is a characteristic of asymptomatic leptospirosis, and we did not assess the excretion of *Leptospira* in our subjects. Thus, further investigations are required.

*Leptospira* serotyping is based on the use of specific monoclonal antibodies, and it allows the distinction of > 300 serovars based on the structural heterogeneity of the surface-exposed lipopolysaccharide^1^. Associations among serovars and animal reservoir hosts have been demonstrated with this method^33^. A recent article showed that a particular molecular type of *Leptospira* is found in a unique ecosystem^1^. Therefore, determining the types of *Leptospira* present in a given area could have great public health, diagnostic, and epidemiological implications. Over 200 *Leptospira* serovars have been identified in Malaysia, mostly in rodents, thereby making them the most important sources of human infections and environmental contamination^34^. In Malaysia, various serovars have been detected in different areas. In Sarawak, reactions to Patoc and Celledoni were the predominant serovars and only nine of the serovars tested were reactive in the samples obtained from planters^35^. In Kelantan, the predominant serovars were reactive to Patoc, Bataviae, and Javanica, and 12 of the tested serovars were reactive in town service workers^36^. The predominant serovars reactive with MAT in the present study were Patoc, Sarawak, and Terengganu, which accounted for more than half of the serum samples tested. Among the 20 serovars tested in our study, 11 serovars were that reacted in MAT, thereby indicating that these serovars might be the specific focus when conducting MAT, which may decrease the workload when performing MAT in the laboratory. Similarly, PCR also obtained positive results for the serum samples that were mainly reactive with the Patoc, Sarawak, and Terengganu serovars by MAT, and they accounted for more than half of the serum samples tested by PCR. Only eight of the 20 serovars tested by MAT were identified by PCR.

The use of personal protective equipment (PPE) and hygiene measures appears to be important for avoiding contact with bacteria, ensuring health protection, and preventing disease^37^. Sanitation workers typically use PPE during their work, but we did not determine the extent of PPE usage among the participants in this study. Overall, the prevalence of *Leptospira* increases as the anthropogenic influence across the landscape increases, accordingly, a significantly higher proportion of infected rodents are observed in urban locations^9^. The survival and persistence of *Leptospira* in the environment depend on its interactions with other microorganisms and reservoir hosts. Recently, an environmental DNA-based approach was developed to identify these factors^38^. Rapid economic development has occurred in Sabah, and it has greatly influenced the urbanization of the area. Thus, an environmental DNA-based approach might be useful for identifying the factors responsible for the environmental survival of *Leptospira* under changing conditions. It is not clear whether these asymptomatic infections are transient or whether appropriate antibiotic treatment is necessary. The long-term consequences of human asymptomatic infections with *Leptospira* need to be explored in details.

In conclusion the high seroprevalence of *Leptospira* among the urban sanitation workers in Kota Kinabalu indicates that a considerable risk is associated with this occupation. Furthermore, a large number of these workers were afflicted with asymptomatic leptospirosis. Because of the nature of their occupation, urban sanitation workers are repeatedly exposed to *Leptospira*, and thus more studies are needed to determine whether asymptomatic leptospirosis is a transient state or whether antibiotic treatment is required. We also determined that a MAT titer < 1:50 indicates the absence of asymptomatic leptospirosis, whereas a MAT titer ≥ 1:50 indicates possible asymptomatic leptospirosis, which should be confirmed by PCR, although a MAT titer of 1:50 can indicate past exposure as well. The seroprevalence of *Leptospira* varies among different areas depending on the *Leptospira* burden, rodent population and other factors, so we advise caution regarding the use of this cutoff value because variations might exist, and it should be independently verified.
